# Synthesis and In Vitro Antitumor Activity of Naringenin Oxime and Oxime Ether Derivatives

**DOI:** 10.3390/ijms20092184

**Published:** 2019-05-02

**Authors:** Ahmed Dhahir Latif, Tímea Gonda, Máté Vágvölgyi, Norbert Kúsz, Ágnes Kulmány, Imre Ocsovszki, Zoltán Péter Zomborszki, István Zupkó, Attila Hunyadi

**Affiliations:** 1 Institute of Pharmacodynamics and Biopharmacy, Interdisciplinary Excellence Centre, University of Szeged, 6720 Szeged, Hungary; latif.ahmed@pharmacognosy.hu (A.D.L.); agnes.kulmany@gmail.com (Á.K.); 2 Institute of Pharmacognosy, Interdisciplinary Excellence Centre, University of Szeged, 6720 Szeged, Hungary; gonda.timea@pharm.u-szeged.hu (T.G.); vagvolgyi.mate@pharm.u-szeged.hu (M.V.); kusz.norbert@pharm.u-szeged.hu (N.K.); zombozope@pharmacognosy.hu (Z.P.Z.); 3 Department of Biochemistry, University of Szeged, 6720 Szeged, Hungary; imre@biochem.szote.u-szeged.hu; 4 Interdisciplinary Centre for Natural Products, University of Szeged, 6720 Szeged, Hungary

**Keywords:** naringenin-oxime, oxime ether, naringenin derivative, antioxidant, cell cycle analysis, antiproliferative, caspase activity

## Abstract

Naringenin is one of the most abundant dietary flavonoids exerting several beneficial biological activities. Synthetic modification of naringenin is of continuous interest. During this study our aim was to synthesize a compound library of oxime and oxime ether derivatives of naringenin, and to investigate their biological activities. Two oximes and five oxime ether derivatives were prepared; their structure has been elucidated by NMR and high-resolution mass spectroscopy. The antiproliferative activity of the prepared compounds was evaluated by MTT assay against human leukemia (HL-60) and gynecological cancer cell lines isolated from cervical (HeLa, Siha) and breast (MCF-7, MDA-MB-231) cancers. *Tert*-butyl oxime ether derivative exerted the most potent cell growth inhibitory activity. Moreover, cell cycle analysis suggested that this derivative caused a significant increase in the hypodiploid (subG1) phase and induced apoptosis in Hela and Siha cells, and induced cell cycle arrest at G2/M phase in MCF-7 cells. The proapoptotic potential of the selected compound was confirmed by the activation of caspase-3. Antioxidant activities of the prepared molecules were also evaluated with xanthine oxidase, DPPH and ORAC assays, and the methyl substituted oxime ether exerted the most promising activity.

## 1. Introduction

Naringenin (**1**) is one of the most abundant dietary flavonoids predominantly found in citrus fruits and grapes. This compound exerts several beneficial pharmacological activities including antioxidant, antiviral, anti-inflammatory, anticarcinogenic and cardioprotective effects [1,2,3]. Several *in vitro* studies showed that naringenin can inhibit cell proliferation and migration, and that it can induce cell cycle arrest and apoptosis in cancer cell lines, including human leukemia, hepatocellular carcinoma, colon, bladder, uterine and breast cancer [4,5,6,7,8,9,10,11]. Due to these biological activities, the design and synthesis of new naringenin derivatives is of continuous interest. With modification of the phenolic groups, several derivatives were synthesized. Esterification and alkylation of the 7-OH group with bulky substituents yielded compounds with improved anticancer effect against human colon cancer cells [12]. Such semi-synthetic modifications also afforded derivatives with significant anti-atherogenic effect in high-cholesterol fed rabbits [13]. Recently it was also found that a 4′- and 7-*O*-methylated naringenin derivative attenuates epileptic seizures in zebrafish and mouse models [14]. Modification on the keto group also yields pharmacologically interesting compounds. Synthetic thiosemicarbazone derivatives were found to exert antioxidative effects and demonstrated significant DNA binding properties [15].

The synthesis and characterization of naringenin *E*-oxime (NOX) has recently been reported. Investigation of its antioxidant and reactive oxygen species (ROS) scavenging properties demonstrated significantly enhanced activity as compared to naringenin itself [16,17]. Further studies confirmed that both naringenin and its *E*-oxime protect cells against hydrogen peroxide-induced oxidative damage [18]. Furthermore, NOX has recently been reported to exert protective effect against cisplatin induced hepatotoxicity, nephrotoxicity and genotoxicity in rats [19]. On the other hand, both naringenin and NOX were found to exert cytotoxic, genotoxic and apoptotic effects by increasing ROS levels in cancer cells, although very high doses were needed (cytotoxic IC_50_ values ranged from ca. 500 to 1000 µM) [20]. Apart from the pharmacological investigations, the chelating capability of NOX makes this compound also interesting from the chemical point of view. The synthesis of Ni(II)-NOX complex was reported, and the prepared ligand was successfully applied in Mizoroki-Heck reaction as a catalyst [21]. In a more recent study, *E*-oximes were prepared from several *O*-alkyl derivatives of naringenin and demonstrated to exert stronger cytotoxic activity on HT-29 cells as compared to their respective parent compounds [22]. However, even though the synthesis and pharmacological investigation of naringenin oxime is reported in several articles [16,17,18,19,20,21], only a few oxime ether derivatives have been mentioned in the literature [23].

During the current work, our aim was to synthesize a set of oxime and oxime ether derivatives of naringenin, and to test their antitumor activity on different cancer cell lines as well as their antioxidant capacity.

## 2. Results and Discussion

### 2.1. Synthesis of Naringenin Oximes and Naringenin Oxime Ethers

Oxime derivatives of racemic naringenin (**1**) were synthesized by using hydroxylamine hydrochloride in the presence of potassium hydroxide and ethanol as solvent. The reaction afforded racemates of two geometric isomers (**2** and **3**), purified by column chromatography and characterized by NMR and HRMS. The ^1^H NMR spectrum of compound **3** displayed resonances of a para disubstituted benzene ring (δ_H_ 7.18 and 6.75, d, *J* = 8.2 Hz), the overlapping signals of two meta coupled aromatic protons (δ_H_ 5.87 br s), and an isolated –CH(O)–CH_2_– spin system (δ_H_ 5.45 br t, *J* = 9.5 Hz; 3.92 dd, *J* = 10.5 Hz, 17.9 Hz; 3.44 dd, *J* = 8.6 Hz, 17.9 Hz). In the ^13^C NMR spectrum 15 carbon resonances were detected, which were then categorized based on their chemical shifts and HSQC cross peaks. The structure of compound **3** was finally established by means of an HMBC experiment. It was obvious that compounds **2** and **3** only differ in the oxime configuration, as suggested by the considerably downfield-shifted C-3 and H-3a/b in compound **3** as compared to **2** (δ_H_ 3.44 dd and 3.92 dd vs. 2.77 dd and 3.25 dd; δ_C_ 45.7 vs. 29.0). Compound **2** was identified as the *E* isomer of naringenin oxime by the literature data [16], thus the minor compound **3** had to be the *Z* isomer. When a ketone is converted into a corresponding oxime derivative, signals of the carbonyl carbon and both adjacent α-carbons shift upfield, and the extent of these changes show a consistent pattern since the α-syn carbons are always more shielded than the α-anti carbons [24]. This structural feature was also clearly seen in compounds **2** and **3**, which further supports the assignation of the two isomers. It is also worth mentioning that the steric hindrance present in compound **3** explains why the formation of the *E* isomer is energetically more favorable. According to HPLC measurements on the crude product mixture, the major and minor products were formed at a ratio of 91:9 during the reaction. Notably, while the preparation of the *E* isomer was discussed in several articles [16,17,18,19,20,21,22,23], this is the first evidence on the formation of the *Z* isomer.

**Figure 1:**
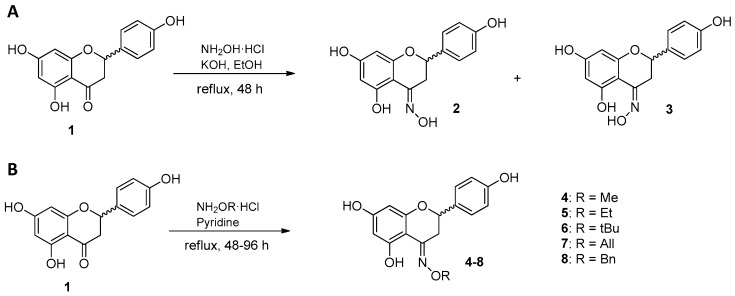
Synthesis of naringenin oximes (**2** and **3**) (**A**) and oxime ethers (**4**–**8**) (**B**).

An oxime ether library (**4**–**8**) was also prepared from naringenin (**1**). Five derivatives were synthesized applying methoxy-, ethoxy-, *tert*-butoxy-, allyloxy-, and benzyloxyamine hydrochloride in pyridine. In all cases, the exclusive formation of the *E* isomer was detected (Figure 1). After purification by flash chromatography, the structures were confirmed by NMR and HRMS techniques.

### 2.2. Biological Activity

#### 2.2.1. Antiproliferative Assay

Antiproliferative activity of naringenin (**1**) and the prepared oximes (**2**, **3**) and oxime ethers (**4**–**8**) was determined in vitro against five human cancer cell lines, including adherent gynecological cell lines isolated from cervical (HeLa, Siha) and breast (MCF-7, MDA-MB-231) carcinomas, and a human leukemia (HL-60) cell line. Two concentrations (25 and 50 μM) were selected for initial bioactivity screening by using an MTT assay, and IC_50_ values were calculated only for those compounds which elicited higher than 75% growth inhibition at 50 μM (Table 1).

Among the tested compounds, the *tert*-butyl substituted derivative (**6**) exerted the most potent antiproliferative effect on the tested cancer cell lines. In contrast, neither naringenin (**1**) nor the *Z*-oxime (**3**) inhibited the growth of the tested cancer cell lines at any of the applied concentrations. In case of the allyl-derivative (**7**) and the *E* oxime (**2**) only a limited growth inhibition was observed. The benzyl-derivative (**8**) exerted moderate growth inhibition on MCF-7 cells, similarly to the methyl derivative (**4**) on HL-60 cells. Our results confirm the previous evidences [20,23] on the increased (but still very weak) antiproliferative activity of naringenin *E*-oxime as compared to that of naringenin (**1**). Further, we show for the first time that the *E*-oxime ethers are much more potent in this regard, particularly if the ether is a bulky alkyl group as in compound **6**. No marked cell line selective action could be demonstrated, though HeLa and MCF-7 cells seem to be more sensitive than SiHa and the triple negative MDA-MB-231 cells.

#### 2.2.2. Cell Cycle Analysis

**Figure 2:**
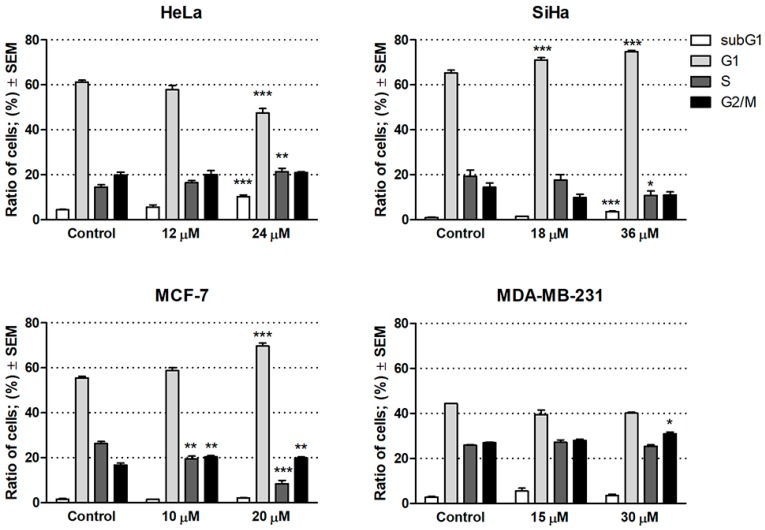
Cell cycle distributions of human gynecological cancer cell lines HeLa, SiHa, MCF-7 and MDA-MB-231 after treatment with compound **6**. *, ** and *** indicate *p* < 0.05, *p* < 0.01 and *p* < 0.001, respectively, by means of one-way ANOVA followed by Dunnett’s post-hoc test.

Based on its outstanding efficacy, compound **6** was selected to subject additional in vitro assays including cell cycle analyses by flow cytometry to characterize the mechanism of action. Cells were treated with compound **6** for 24 h at concentrations corresponding to its half IC_50_ or IC_50_ values, i.e., 12 or 24 μM for HeLa, 18 or 36 µM for SiHa, 10 or 20 µM for MCF-7, and 15 or 30 µM for MDA-MB-231 (Figure 2).

A significant increase in the hypodiploid (subG1) phase was found only in HeLa and SiHa cells after the treatment with the higher concentrations (24 and 36 µM, respectively), indicating the induction of apoptosis in these cell lines. In addition, SiHa exhibited significant accumulation of cells in G1 phase with a corresponding decrease in the fraction of cells in the S phase, which may be a consequence of blockade of the G1–S transition of the cell cycle. In HeLa cells, however, significant decrease was observed in the G1 phase accompanied by a significant increase in the S phase. In case of the MCF-7 cells, the proportion of cells in the G1 and G2/M phase increased, whereas the proportion in the S phase decreased, suggesting cell cycle arrest at G1 and G2/M phase. On the other hand, no substantial action was observed on MDA-MB-231 cells at 15 and 30 µM concentrations after 24 h of treatment. Our results indicate that compound **6** induced apoptosis in HeLa and Siha cell lines and exerted a disturbance in the cell cycle.

#### 2.2.3. Caspase Activity

**Figure 3:**
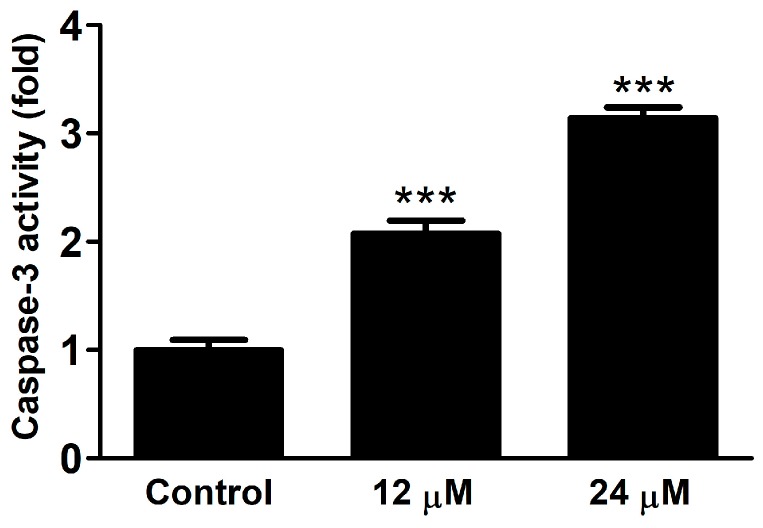
Effect of compound 6 on the caspase-3 activity in HeLa cells as compared to the untreated control. Results are means ± standard error of the mean from 5 replicates. *** indicates *p* < 0.001, by means of one-way ANOVA followed by Dunnett’s post-hoc test.

As the *tert*-butyl derivative (**6**) exerted pronounced antiproliferative activity by inducing apoptosis, its effect on caspase-3 activity was also tested. HeLa cells were treated for 24 h with compound **6** in two different concentrations (12 and 24 μM). Compound **6** induced a concentration-dependent and significant increase of caspase-3 activity (Figure 3).

#### 2.2.4. Antioxidant Activity

Antioxidant activity of naringenin (**1**) and its oxime derivatives (**2**–**8**) was analyzed through assessing their capacity to scavenge diphenyl-2-picrylhydrazyl (DPPH) radical and determining their oxygen radical absorbance capacity (ORAC) and xanthine-oxidase (XO) inhibitory activity (Table 2).

Previously, Türkkan et al. showed that the 4-oxime substitution increases the antioxidant activity of naringenin [16], which here we can confirm concerning the DPPH scavenging activity. However, it is now also clear that a favorable increase in this activity can only be observed for the *E*-oxime (**2**). While it is still stronger DPPH scavenger than naringenin (**1**), the Z-oxime (**3**) was an order of magnitude weaker in this regard than the *E*-oxime (**2**). However, regardless of the orientation of the oxime, the ORAC activity of both compounds **2** and **3** was decreased as compared to naringenin (**1**). Surprisingly, the oxime methyl ether derivative (**4**) showed the highest antioxidant activity in both the DPPH and the ORAC assays, and this was the only compound that was more potent in the ORAC assay than the positive control rutin. Since rutin has a catechol-type B-ring that makes it a particularly efficient ROS scavenger, this finding suggests that an oxime methyl ether moiety on the flavanone C-ring is highly favorable in terms of such activity. Accordingly, it may be hypothesized that the 4-oxime methyl ether of eriodictyol (the catechol B-ring analog of naringenin) would be an even stronger antioxidant in vitro. All other compounds, including the *tert*-butyl derivative **6**, exhibited much weaker antioxidant activity than rutin, and they were also proven practically inactive in terms of XO inhibition.

Structure-activity relationships observed for the antioxidant activity of naringenin oxime derivatives did not show any apparent correlation to those of the in vitro antiproliferative activity, suggesting that the antitumor potential of these compounds is likely not, or at least not entirely due to their antioxidant properties. Further studies are needed to reveal the exact mechanism of action. Nevertheless, it is now clear that the introduction of a bulky alkyl (e.g., *tert*-butyl) group as an oxime ether to the flavanone B-ring greatly increases the antitumor potential of such compounds, which finding shows new paths towards the design and optimization of new, bioactive flavonoid analogs.

## 3. Materials and Methods

### 3.1. Chemical Methods

Starting material naringenin (purity: 98%) was purchased from Indofine Chemical Company Inc. (Hillsborough, NJ, USA), and used without further purification. All reagents were purchased from Sigma (Sigma Aldrich Co., St. Louis, MO, USA). Ethanol 96% was obtained from Molar Chemicals Ltd. (Halásztelek, Hungary). Reaction progress was monitored by normal phase thin-layer chromatography (TLC) (Silica gel 60F254, E. Merck, Darmstadt, Germany). Purification was carried out applying flash chromatography on a CombiFlash Rf+ Lumen Instrument (Teledyne Isco, Lincoln, NE, USA) with integrated UV-Vis, photodiode-array (PDA) and evaporative light-scattering (ELS) detection on commercially available pre-filled columns (Teledyne Isco, Lincoln, NE, USA) for normal-phase separations in a detection range of 210–366 nm. Naringenin oximes and oxime ethers were characterized by means of NMR and MS. ^1^H (500.1 MHz) and ^13^C (125.6 MHz) NMR spectra were recorded on a Bruker Avance DRX-500 spectrometer (Bruker, Billerica, MA, USA).

### 3.2. Cell Culture

All cell lines were obtained from ECCAC (European Collection of Cell Cultures, Salisbury, UK) except the SiHa and human leukemia cells HL-60, which were obtained from ATCC (American Tissue Culture Collection, Manassas, VA, USA). The human gynecological cancer cell lines were cultivated in minimal essential medium (MEM) supplemented with 10% fetal bovine serum, 1% non-essential amino acids and an antibiotic–antimycotic mixture. HL-60 cells were grown in RPMI 1640 medium containing 10% heat inactivated fetal calf serum (FCS), 1% L-glutamine, and 1% penicillin-streptomycin. The cell lines were cultured at 37 °C in a humidified atmosphere 5% CO_2_. All media and chemicals used in our experiment, if otherwise not specified, were purchased from from Lonza Group Ltd. (Basel, Switzerland) and Sigma-Aldrich Ltd. (Budapest, Hungary). [25,26].

### 3.3. Antiproliferative Assay

Inhibitory growth effects of the compounds evaluated on human leukemia cells (HL-60) and human gynecological adherent cancer cell lines (HeLa, SiHa, MCF-7, MDA-MB-231) isolated from cervical and breast cancer has been carried out according to our previously published procedures [27,28] by using MTT ([3-(4,5 dimethylthiazol-2-yl)-2,5-diphenyltetrazolium bromide]) assay. Briefly, adherent cells were seeded in 96-well microplates (5000 cells/well) and overnight preincubation allowed attachment to the bottom of the well before treatment, while the HL-60 cells seeded at 10,000 cells/well and treated the same day. Thereafter, 50 mM of the compounds were dissolved in dimethyl sulfoxide (DMSO), and the cells were treated with two concentrations (25 and 50 µM) and incubated for 72 h. Then the solution of 5 mg/mL MTT was added to the samples and incubated for another 4 h. For adherent cancer cell lines, the medium was removed, and the precipitated crystals were dissolved by adding 100 µL DMSO under stirring for 1 h, at 37 °C and the absorbance was read at 545 nm, using a microplate reader. In the case of leukemia cells, the precipitated crystals were dissolved in 10% sodium dodecyl sulfate with acid HCl 0.01 mM, and incubated for 24 h, the absorbance was read at 545 and 630 nm. The determination of the antiproliferative compound was repeated with a set of dilutions (1–50 µM) to calculate the IC_50_ value. Calculations and statistical analyses (One-Way ANOVA followed by Dunnett’s post-hoc test) were performed by Graph Pad Prism 5.01 (Graph Pad Software; San Diego, CA, USA). All measurements in all experiments were carried out in duplicate reading with at least five parallel wells.

### 3.4. Cell Cycle Analysis

Estimation of cellular DNA content has been carried out using flow cytometric analysis with DNA-specific fluorescent dye, propidium iodide (PI) as published before [29]. Briefly, adherent cancer cells were plated in a 6-well plate at a density of 400,000 cells/well, and allowed to grow for 24 h, cells were treated with the compound in two concentrations: 12 to 24 µM for Hela; 18 to 36 µM for Siha; 15 to 30 µM for MDA-MB-231 and 10 to 20 µM for MCF-7 with incubation period of 24 h. The cells were washed with phosphate-buffered saline (PBS), following dissociation with trypsin, cells were centrifuged at 2200 rpm, 4 °C for 15 min. After the washing step, the cells were fixed in 1 mL 70% cold ethanol which was added dropwise to the cell pellet and the cells were kept at −20 °C until DNA staining. Prior to analysis, the cells were stained with 300 µL dye solution containing 0.02 mg/mL RNAse A, 0.1 mg/mL PI, 0.003 mL/mL Triton-X and 1.0 mg/mL sodium citrate in distilled water, incubated in the dark place for 60 min at room temperature. Finally, 700 µL PBS was added and mixed to the sample. Flow cytometric analyses were carried out using a flow cytometer PartecCyFlow instrument (Partec GmbH, Münster, Germany). In each analysis, 20,000 events were counted, and ModFit Software (Verity Software House, Topsham, ME, USA) was used to determine the different percentages of cell cycle phases (subG1, G1, S and G2/M). The results were statistically evaluated by Graph Pad Prism 5.0. (GraphPad Software Inc., San Diego, CA, USA) using one-way ANOVA for two biological replicates.

### 3.5. Caspase Activity

Caspase-3 activity was determined by using Caspase-3 Colorimetric Assay Kit as published before [30]. Briefly, HeLa cells were seeded at 12 million cells /flask density and allowed to grow overnight. The cells were treated with compound 6 and incubated for 24 h. Then they were scraped, centrifuged and washed with physiological buffer saline, re-suspended in lysis buffer (1 million cells/10 µL) and incubated on ice for 20 min, then cold centrifuged (16,000× *g*, 4 °C for 15 min). Then the supernatant was used for measurement of caspase-3 activity. The concentration of the protein was measured with the substrate Assay Kit, by incubating 5.0 µL of treated and untreated lysates samples with 10 µL selective caspase-3 substrate in a final volume of 100 µL in assay buffer, and for the experiment control 5.0 µL of 24 µM sample was added with 10 µL caspase-3 substrate and 10 µL caspase-3 inhibitor, in a final volume of 100 µL in assay buffer in 96-well plates. After an incubation period of 24 h at 37 °C, 5% CO_2_, the absorbance was measured at 405 nm with a microplate reader. The results were statistically evaluated by Graph Pad Prism 5.0. using one-way ANOVA.

### 3.6. Antioxidant Activity

#### 3.6.1. Diphenyl-2-picrylhydrazyl (DPPH) Assay

DPPH (1,1′-diphenyl-2-picrylhydrazyl) was purchased from Sigma-Aldrich Hungary. DPPH free radical scavenging assay was performed according to the method of Fukomoto et al. [31] with some modifications. The measurement was carried out on a 96-well microplate. Microdilution series of samples (10 mM stock solution, dissolved in DMSO) were made starting with 150 µL. To each well 50 µL of DPPH reagent (100 µM in HPLC grade MeOH) was added to gain 200 µL working volume. The microplate was stored at room temperature in dark conditions. The absorbance was measured after 30 min at 550 nm using a FluoStar Optima plate reader (software version 2.20R2, BMG Labtech Ortenberg, Germany). For the blank control DMSO was used instead of the sample. As a standard, rutin (0.01 mg/mL, in HPLC grade MeOH) was used. The scavenging activity was calculated as Inhibition (%) = (A_0_ − A_s_)/A_0_ × 100, and EC_50_ values were calculated by Graph Pad Prism 6.05 software.

#### 3.6.2. Oxygen Radical Absorbance Capacity (ORAC) Assay

AAPH ((2,2′-Azobis(2-methyl-propionamidine) dihydrichloride) free radical and trolox standard ((±)-6hydroxy-2,5,7,8-tetramethyl-chromane-2-carboxylic acid) were purchased from Sigma-Aldrich Hungary. Fluorescein was purchased from Fluka Analytical, Tokyo, Japan. The ORAC assay was carried out on a 96-well microplate according to the method of Mielnik et al. [32]. Briefly, 20 µL of extracts (stock solution concentration of 0.002 mM) were mixed with 60 µL of AAPH (12 mM final concentration) and 120 µL of fluorescein solution (70 nM final concentration), then the fluorescence was measured through 3 h with 1.5-min cycle intervals with a BMG Labtech FluoStar Optima plate-reader. All experiments were carried out in triplicate, and trolox was used as standard. The antioxidant capacity was expressed as µM Trolox Equivalent per μM of pure compound (µM TE/μM), as calculated by Graph Pad Prism 6.05.

#### 3.6.3. Xantine-Oxidase Inhibitory Assay

To assess xanthine-oxidase (XO) inhibitory activity, a continuous spectrophotometric rate determination was used, based on a modified protocol of Sigma. The absorbance of XO-induced uric acid production from xanthine was measured at 290 nm for 3 min in a 96-well plate on a BMG Labtech FluoStar Optima plate-reader. The XO-inhibitory effect was determined via the decreased production of uric acid. The samples (10 mM stock solution) were prepared in DMSO. For enzyme-activity control, the final reaction mixture comprised of 100 μL of xanthine solution (0.15 mM, pH = 7.5), 150 μL of buffer (potassium phosphate with 1 M KOH, pH = 7.5) and 50 μL of XO (0.2 units/mL) in a 300 μL well. The reaction mixture for inhibition was made with 100 μL of xanthine, 140 μL of buffer, 10 μL of sample and 50 μL of XO. Allopurinol was used as positive control. Test compound samples were added in appropriate volumes so that the final concentration of DMSO in the assay did not exceed 3.3% of the total volume. All experiments were conducted in triplicates. The reaction was initiated by the automatic addition of 50 µL of XO solution to a final concentration of 0.006 units/mL. The inhibitory percentage values were calculated by using Graph Pad Prism 6.05 software.

## 4. Experimental

### 4.1. Procedure for the Synthesis of Naringenin-Derived Oximes

Naringenin (1.00 g) was dissolved in 100 mL EtOH, then 3 equiv. of KOH (0.62 g) and 3 equiv. of hydroxylamine hydrochloride (0.77 g) were added. The reaction mixture was refluxed for 48 h, then the solvent was evaporated under vacuo. The residue was re-dissolved in water (100 mL) and the aqueous phase was extracted with EtOAc (3 × 100 mL). The organic phase was combined and dried (Na_2_SO_4_), then evaporated to dryness. The crude product was purified by flash chromatography using *n*-hexane—EtOAc—formic acid (15:4:0.25, *v*/*v*/*v*) solvent system on silica.

(E)-5,7-dihydroxy-2-(4-hydroxyphenyl)chroman-4-one oxime (**2**)

Yield: 38%, white solid. ^1^H NMR (500 MHz, DMSO-*d*_6_): δ 11.23 (1H, s), 11.20 (1H, br s), 9.80 (1H, br s), 9.53 (1H, br s), 7. 28 (2H, d, *J* = 8.4 Hz), 6.78 (2H, d, *J* = 8.4 Hz), 5.90 (1H, d, *J* = 1.8 Hz), 5.85 (1H, d, *J* = 1.8 Hz), 5.02 (1H, dd, *J* = 2.5 Hz, 11.5 Hz), 3.25 (1H, dd, *J* = 2.7 Hz, 17.0 Hz), 2.77 (11.7 Hz, 17.0 Hz). ^13^C NMR (MHz, DMSO-*d*_6_): δ 160.3, 159.1, 157.9, 157.5, 153.3, 129.9, 128.0, 115.1, 97.0, 96.4, 95.2, 75.8, 29.0 HR-HESI-MS C_15_H_14_NO_5_ [M + H]^+^ calcd. 288.0871, found: 288.0884.

(Z)-5,7-dihydroxy-2-(4-hydroxyphenyl)chroman-4-one oxime (**3**)

Yield: 3%, white solid. ^1^H NMR (500 MHz, DMSO-*d*_6_): δ 10.25 (2H, br s), 9.75 (1H, br s), 9.50 (1H, br s), 7.18 (2H, d, *J* = 8.2 Hz), 6.75 (2H, d, *J* = 8.2 Hz), 5.87 (2H, s), 5.45 (1H, br t, *J* = 9.5 Hz), 3.92 (1H, dd, *J* = 10.5 Hz, 17.9 Hz), 3.44 (1H, dd, *J* = 8.6 Hz, 17.9 Hz). ^13^C NMR (MHz, DMSO-*d*_6_): δ 160.7, 158.9, 157.8, 157.3, 153.9, 130.7, 127.7, 115.3, 95.3, 94.7, 80.2, 45.7. HR-HESI-MS C_15_H_14_NO_5_ [M + H]^+^ calcd. 288.0871, found: 288.0888.

### 4.2. General Procedure for the Synthesis of Naringenin Oxime Ethers

To begin with, 100 mg naringenin was dissolved in 15 mL pyridine, then 3 equiv. of the corresponding alkyl or aryloxyhydroxylamine hydrochloride were added and the mixture was refluxed for 48–96 h. When the reaction was completed (monitored by means of TLC), the solvent was evaporated under vacuo. Water (50 mL) was added to the residue and the aqueous phase was extracted with EtOAc (3 × 50 mL). The combined organic phase was dried over Na_2_SO_4_, filtered and evaporated to dryness. Each crude product was purified with flash chromatography on silica.

(E)-5,7-dihydroxy-2-(4-hydroxyphenyl)chroman-4-one *O*-methyl oxime (**4**)

Purified by using *n*-hexane—EtOAc—formic acid (15:4:0.25, *v*/*v*/*v*), yield: 18%, white solid. ^1^H NMR (500 MHz, DMSO-*d*_6_): δ10.71 (1H, s), 9.98 (1H, br s), 9.58 (1H, br s), 7.27 (2H, d, *J* = 8.3 Hz), 6.77 (2H, d, *J* = 8.3 Hz), 5.92 (1H, br s), 5.87 (1H, br s), 5.03 (1H, dd, *J* = 2.4 Hz, 11.9 Hz), 3.88 (3H, s), 3.23 (1H, dd, *J* = 2.4 Hz, 17.1 Hz), 2.82 (1H, dd, *J* = 11.9 Hz, 17.1 Hz). ^13^C NMR (MHz, DMSO-*d*_6_): δ 161.1, 159.1, 158.4, 157.5, 154.4, 129.6, 128.1, 115.2, 96.5, 96.2, 95.5, 75.6, 62.1, 29.5. HR-HESI-MS C_16_H_16_NO_5_ [M + H]^+^ calcd. 302.1028, found: 302.1023.

(E)-5,7-dihydroxy-2-(4-hydroxyphenyl)chroman-4-one *O*-ethyl oxime (**5**)

Purified by dichloromethane—methanol (99:1, *v*/*v*), yield: 52%, white solid. ^1^H NMR (500 MHz, DMSO-*d*_6_): δ 10.82 (1H, s), 9.94 (1H, br s), 9.55 (1H, br s), 7.28 (2H, d, *J* = 8.5 Hz), 6.77 (2H, d, *J* = 8.5 Hz), 5.92 (1H, d, *J* = 2.2 Hz). 5.87 (1H, d, *J* = 2.2 Hz), 5.03 (1H, dd, *J* = 2.7 Hz, 11.9 Hz), 4.13 (2H, m), 3.23 (1H, dd, *J* = 2.7 Hz, 17.1 Hz), 2.83 (1H, dd. *J* = 11.9 Hz, 17.1 Hz), 1.24 (3H, t, *J* = 7.0 Hz).^13^C NMR (MHz, DMSO-*d*_6_): δ 160.9, 159.1, 158.4, 157.6, 154.1, 129.6, 128.2, 115.1, 96.5, 96.4, 96.4, 75.7, 69.6, 29.6, 14.2. HR-HESI-MS C_17_H_18_NO_5_ [M + H]^+^ calcd. 316.1185, found: 316.1181.

(E)-5,7-dihydroxy-2-(4-hydroxyphenyl)chroman-4-one *O*-(tert-butyl) oxime (**6**)

Purified by dichloromethane—methanol (99:1, *v*/*v*), yield: 53%, white solid. ^1^H NMR (500 MHz, DMSO-*d*_6_): δ 11.10 (1H, s), 9.90 (1H, br s), 9.55 (1H, br s), 7.28 (2H, d, *J* = 8.5 Hz), 6.77 (2H, d, *J* = 8.5 Hz), 5.91 (1H, d, *J* = 2.2 Hz). 5.86 (1H, d, *J* = 2.2 Hz), 5.03 (1H, dd, *J* = 2.6 Hz, 11.9 Hz), 3.21 (1H, dd, *J* = 2.6 Hz, 17.1 Hz), 2.82 (1H, dd, *J* = 11.9 Hz, 17.1 Hz), 1.29 (9H, s). ^13^C NMR (MHz, DMSO-*d*_6_): δ160.7, 159.1, 158.3, 157.6, 153.5, 129.7, 128.2, 115.1, 96.9, 96.4, 95.4, 78.9, 75.9, 29.6, 27.1. HR-HESI-MS C_19_H_22_NO_5_ [M + H]^+^ calcd. 344.1498, found: 344.1496.

(E)-5,7-dihydroxy-2-(4-hydroxyphenyl)chroman-4-one *O*-allyl oxime (**7**)

Purified by dichloromethane—methanol (95:5, *v*/*v*), yield: 58%, white solid. ^1^H NMR (500 MHz, DMSO-*d*_6_): δ 10.72 (1H, s), 9.95 (1H, br s), 9.54 (1H, br s), 7.28 (2H, d, *J* = 8.5 Hz), 6.77 (2H, d, *J* = 8.5 Hz), 5.99 (1H, m), 5.91 (1H, d, *J* = 2.2 Hz), 5.86 (1H, d, *J* = 2.2 Hz), 5.35 (1H, dd, *J* = 1.3 Hz, 17.3 Hz), 5.25 (1H, br d, *J* = 10.4 Hz), 5.05 (1H, dd, *J* = 2.7 Hz, 11.8 Hz), 4.60 (2H, d, *J* = 5.7 Hz), 3.25 (1H, dd, *J* = 2.7 Hz, 17.1 Hz), 2.86 (1H, dd, *J* = 11.8 Hz, 17.1 Hz).^13^C NMR (MHz, DMSO-*d*_6_): δ 161.0, 159.1, 158.4, 157.5, 154.4, 134.1, 129.6, 123.1, 118.5, 115.1, 96.5, 96.4, 95.4, 75.7, 74.6, 29.6. HR-HESI-MS C_19_H_22_NO_5_ [M + H]^+^ calcd. 344.1498, found: 344.1496.

(E)-5,7-dihydroxy-2-(4-hydroxyphenyl)chroman-4-one *O*-benzyl oxime (**8**)

Purified by dichloromethane—methanol (99:1, *v*/*v*): yield: 5%, white solid. ^1^H NMR (500 MHz, DMSO-*d*_6_): δ 10.63 (1H, s), 9.94 (1H, s), 9.53 (1H, s), 7.42–7.32 (5H, m), 7.26 (2H, d, *J* = 8.4 Hz), 6.76 (2H, d, *J* = 8.4 Hz), 5.87 (1H, d, *J* = 2.2 Hz), 5.85 (1H, d, *J* = 2.2 Hz), 5.14 (2H, s), 5.04 (1H, dd, *J* = 2.9 Hz, 11.7 Hz), 3.25 (1H, dd, *J* = 3.0 Hz, 17.1 Hz), 2.90 (1H, dd, *J* = 11.7 Hz, 17.1 Hz). ^13^C NMR (MHz, DMSO-*d*_6_): δ 161.0, 159.0, 158.3, 157.5, 154.6, 137.2, 129.5, 128.4, 128.4 128.1, 128.0, 115.1, 96.5, 96.3, 95.4, 75.6, 75.6, 29.5. HR-HESI-MS C_22_H_20_NO_5_ [M + H]^+^ calcd. 378.1341, found: 378.1342.

## Figures and Tables

**Table 1:** Antiproliferative activities of naringenin and its oxime derivatives against four human gynecological cancer cell lines and HL-60 leukemia cells. Cisplatin was used as positive control; SEM: standard error of the mean; *n* = 5.

Compound	Conc. (µM)	Growth Inhibition (%) ± SEM (Calculated IC_50_ Value (µM))				
		HeLa	SiHa	MCF-7	MDA-MB-231	HL-60
(**1**)	25	<20	<20	<20	<20	<20
	50	23.9 ± 2.09	<20	<20	<20	<20
(**2**)	25	<20	<20	<20	<20	<20
	50	28.75 ± 2.44	<20	21.83 ± 3.92	<20	43.40 ± 2.81
(**3**)	25	<20	<20	<20	<20	<20
	50	<20	<20	<20	<20	<20
(**4**)	25	<20	<20	<20	<20	37.67 ± 1.29
	50	31.36 ± 2.97	<20	48.44 ± 3.27	24.35 ± 1.88	57.89 ± 1.13
(**5**)	25	<20	<20	<20	<20	<20
	50	29.36 ± 1.42	<20	44.06 ± 2.18	<20	44.89 ± 0.48
(**6**)	25	52.37 ± 2.32	<20	61.41 ± 1.93	27.19 ± 1.78	37.31 ± 3.65
	50	92.22 ± 1.03	88.54 ± 1.51	87.00 ± 0.61	90.33 ± 0.58	88.07 ± 0.10
		[23.49]	[35.41]	[19.46]	[29.74]	[31.76]
(**7**)	25	<20	<20	<20	<20	<20
	50	25.04 ± 2.4	<20	33.75 ± 2.45	<20	<20
(**8**)	25	22.63 ± 0.63	<20	24.29 ± 1.86	<20	<20
	50	37.67 ± 2.01	<20	64.47 ± 2.12	24.87 ± 3.47	<20
**Cisplatin**	25	98.71 ± 0.21	86.40 ± 1.02	90.81 ± 0.22	41.37 ± 1.05	64.03 ± 0.43
	50	99.09 ± 0.24	96.72 ± 0.36	98.49 ± 0.11	84.43 ± 0.4	84.88 ± 0.41
		[11.79]	[13.63]	[5.15]	[25.82]	[5.75]

**Table 2:** Antioxidant activity of compounds **1**–**8**. TE: trolox equivalent; XO inh: xanthine oxidase inhibition. ^a^ Compounds eliciting less than 50% scavenging of diphenyl-2-picrylhydrazyl (DPPH) at the highest applied concentration were considered inactive and the numerical results are not presented; ^b^ inhibition % at 330 µM, n.d.: not determined; SD: standard deviation.

Compound	Antioxidant Activity ± SD		
	DPPH EC_50_ (μM)	ORAC (μmolTE/μmol)	XO inh (%)
**1**	- ^a^	11.18 ± 0.46	12.31 ± 4.60 ^b^
**2**	243.45 ± 4.88	8.88 ± 0.23	7.35 ± 1.32
**3**	1776.00 ± 123.71	6.95 ± 0.12	2.13 ± 0.78
**4**	212.20 ± 32.59	16.63 ± 1.68	4.00 ± 1.81
**5**	1437.50 ± 36.06	5.54 ± 0.41	8.13 ± 2.02
**6**	-	3.89 ± 0.87	6.95 ± 2.31
**7**	1164.00 ± 226.27	6.03 ± 2.79	12.84 ± 3.01
**8**	-	1.38 ± 0.41	9.06 ± 0.79
**Rutin**	39.88 ± 1.34	12.35 ± 0.38	n.d.
**Allopurinol**	n.d.	n.d.	98.23 ± 3.29
